# *CHCHD10* mutations induce tissue-specific mitochondrial DNA deletions with a distinct signature

**DOI:** 10.1093/hmg/ddad161

**Published:** 2023-10-10

**Authors:** Mario K Shammas, Yu Nie, Alexandra Gilsrud, Xiaoping Huang, Derek P Narendra, Patrick F Chinnery

**Affiliations:** Department of Clinical Neurosciences, School of Clinical Medicine, University of Cambridge, Cambridge Biomedical Campus, Cambridge CB2 0QQ, United Kingdom; Medical Research Council Mitochondrial Biology Unit, University of Cambridge, Cambridge Biomedical Campus, Hills Road, Cambridge CB2 0XY, United Kingdom; Inherited Movement Disorders Unit, Neurogenetics Branch, National Institute of Neurological Disorders and Stroke, National Institutes of Health, 35 Convent Drive, Bethesda, MD 20892, United States; Department of Clinical Neurosciences, School of Clinical Medicine, University of Cambridge, Cambridge Biomedical Campus, Cambridge CB2 0QQ, United Kingdom; Medical Research Council Mitochondrial Biology Unit, University of Cambridge, Cambridge Biomedical Campus, Hills Road, Cambridge CB2 0XY, United Kingdom; Inherited Movement Disorders Unit, Neurogenetics Branch, National Institute of Neurological Disorders and Stroke, National Institutes of Health, 35 Convent Drive, Bethesda, MD 20892, United States; Inherited Movement Disorders Unit, Neurogenetics Branch, National Institute of Neurological Disorders and Stroke, National Institutes of Health, 35 Convent Drive, Bethesda, MD 20892, United States; Inherited Movement Disorders Unit, Neurogenetics Branch, National Institute of Neurological Disorders and Stroke, National Institutes of Health, 35 Convent Drive, Bethesda, MD 20892, United States; Department of Clinical Neurosciences, School of Clinical Medicine, University of Cambridge, Cambridge Biomedical Campus, Cambridge CB2 0QQ, United Kingdom; Medical Research Council Mitochondrial Biology Unit, University of Cambridge, Cambridge Biomedical Campus, Hills Road, Cambridge CB2 0XY, United Kingdom

**Keywords:** mitochondria, neurodegeneration, mtDNA deletions, mitochondrial DNA, CHCHD10

## Abstract

Mutations affecting the mitochondrial intermembrane space protein CHCHD10 cause human disease, but it is not known why different amino acid substitutions cause markedly different clinical phenotypes, including amyotrophic lateral sclerosis-frontotemporal dementia, spinal muscular atrophy Jokela-type, isolated autosomal dominant mitochondrial myopathy and cardiomyopathy. *CHCHD10* mutations have been associated with deletions of mitochondrial DNA (mtDNA deletions), raising the possibility that these explain the clinical variability. Here, we sequenced mtDNA obtained from hearts, skeletal muscle, livers and spinal cords of WT and Chchd10 G58R or S59L knockin mice to characterise the mtDNA deletion signatures of the two mutant lines. We found that the deletion levels were higher in G58R and S59L mice than in WT mice in some tissues depending on the Chchd10 genotype, and the deletion burden increased with age. Furthermore, we observed that the spinal cord was less prone to the development of mtDNA deletions than the other tissues examined. Finally, in addition to accelerating the rate of naturally occurring deletions, Chchd10 mutations also led to the accumulation of a novel set of deletions characterised by shorter direct repeats flanking the deletion breakpoints. Our results indicate that Chchd10 mutations in mice induce tissue-specific deletions which may also contribute to the clinical phenotype associated with these mutations in humans.

## Introduction

Mitochondria generate energy for the cell through oxidative phosphorylation (OXPHOS) [1]. While most of the mitochondrial proteome is encoded by the nucleus, mitochondria additionally have their own mitochondrial DNA (mtDNA) which encodes for 13 subunits of the electron transport chain and ATP synthase, integral for OXPHOS [2–4]. Each mitochondrion contains 2–10 mtDNA molecules, and therefore a cell’s mtDNA copy number (CN) is directly proportional to the number of mitochondria contained in the cell, ranging from hundreds to thousands of copies. In contrast to nuclear DNA (nDNA), mtDNA is circular, lacks introns and replicates independently of the cell cycle (reviewed by Rackham and Filipovska [5]).

MtDNA in different tissues tends to accumulate large-scale deletions with age, with these deletions possibly contributing to the phenotypes associated with aging [6–10]. Although the causes of mtDNA deletions are not well-understood, studies targeting restriction enzymes to the mitochondrial matrix showed that double-stranded breaks (DSBs) could lead to the formation of mtDNA deletions [11, 12]. Additionally, when examining the breakpoints of naturally occurring deletions, it was observed that deletions are mostly found in the major arc and are commonly flanked by direct repeats between 8–13 bp long [13–16]. This, along with the observation that the repeat closest to the origin of replication of the light strand (OriL) tended to be retained after the deletion, led to the development of the copy-choice recombination hypothesis of deletion formation, whereby, if polymerase γ temporarily dissociates from the template DNA during synthesis of the first repeat, the 3′-end of the newly-synthesised light strand dissociates from the first direct repeat on the heavy strand and aberrantly binds the second direct repeat closer to OriH [17]. Upon a second round of replication, this results in a full length mtDNA molecule and a mtDNA molecule with a deletion in the region between the repeats and including the repeat closer to OriH.

Mutations in several nuclear-encoded mitochondrial proteins have been found to cause multiple mtDNA deletion syndromes (reviewed by Viscomi and Zeviani [18]). These proteins generally fall into one of three categories: (1) proteins involved in replicating or maintaining mtDNA (e.g. POLG, TWNK and MGME1); (2) proteins involved in nucleotide synthesis or import into the matrix (e.g. ANT1, TK2 and TYMP); or (3) proteins regulating mitochondrial dynamics (e.g. OPA1 and MFN2) [19–27]. While the first two categories are directly involved in maintaining mtDNA or supplying an adequate nucleotide pool for replication, it is not clear how altering mitochondrial dynamics affects mtDNA stability.

In the last decade, mutations affecting the mitochondrial intermembrane space (IMS) protein coiled-coil-helix-coiled-coil-helix domain-containing 10 (CHCHD10) have been identified as causing multiple mtDNA deletions [28]. Autosomal dominant mutations in *CHCHD10* and its paralog *CHCHD2* lead to a wide spectrum of neurodegenerative and neuromuscular diseases, including amyotrophic lateral sclerosis-frontotemporal dementia (ALS-FTD, CHCHD10^S59L^), ALS (CHCHD10^R15L^), spinal muscular atrophy Jokela-type (SMA-J, CHCHD10^G66V^), autosomal dominant isolated mitochondrial myopathy and cardiomyopathy (IMMD, CHCHD10^G58R^) and Parkinson’s disease (CHCHD2^T61I^) [29–39]. Although their functions are yet to be established, CHCHD2 and CHCHD10 proteins exhibit partial functional redundancy, and loss of both proteins results in a mild OXPHOS deficit and altered cristal structure [40, 41]. The pronounced effects of the patient mutations therefore likely arise through a toxic gain of function mechanism [36, 42, 43].

Chchd10^S59L/+^ mice (S55L in mouse, but we will use the human S59L notation for consistency) were previously generated and found to recapitulate the myopathic phenotype of the family [42, 43]. The mice additionally exhibited a cardiomyopathy (not seen in humans with that mutation) that was fatal by 14 months of age. Although ALS-FTD was an important feature in humans [35], Chchd10^S59L^ mice did not exhibit prominent CNS involvement, with minimal spinal cord motor neuron loss and no brain neurodegeneration observed [42, 43]. We additionally previously generated a Chchd10^G58R/+^ mouse (G54R mutation in mouse, but we will use the human G58R notation for consistency) which recapitulates the myopathy and cardiomyopathy of the family harbouring that mutation [36]. Although previous work showed that mutations in *CHCHD10* lead to multiple mtDNA deletions [35, 36, 42, 43], it is not known whether these are due to an acceleration of age-related deletions (as we previously observed in anti-retroviral therapy [44]) or due to novel deletions specific to this disease. Additionally, it is not known whether pathogenic *CHCHD10* mutations cause increased mtDNA single nucleotide variant (SNV) mutational burden.

In this work, we sequenced mtDNA obtained from hearts, skeletal muscle, livers and spinal cords of WT, G58R and S59L mice to characterise the mtDNA deletion signatures of the two mutant lines.

## Results

**Figure 1:**
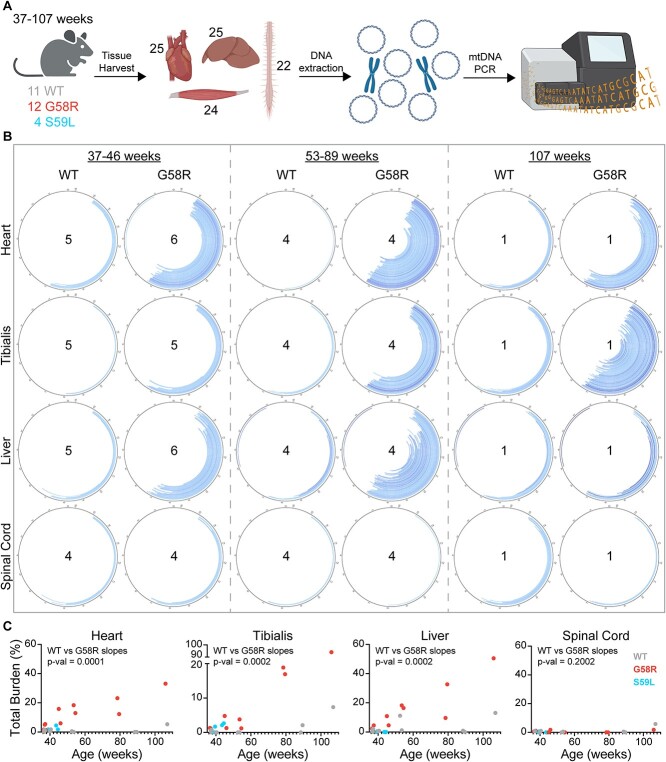
Deletions in WT and G58R mice. (A) Outline of the experimental setup. Tissue was harvested from WT, G58R and S59L mice (25 hearts, 25 livers, 24 tibialis anterior muscles and 22 spinal cords). Whole DNA was extracted from the tissue samples and mtDNA was amplified by PCR and sequenced. (B) Circle plots representing mtDNA and showing the deletions called through MitoSAlt. Darker shades correspond to higher heteroplasmy (range: 0.5–16%). Deletions shown are compiled from the number of samples indicated in the centre of the circle. (C) Total deletion burden vs. age plots. Each dot indicates a different sample. For (C) an equality of slopes test was performed for WT vs G58R.

To better understand the landscape of mtDNA alterations caused by Chchd10 mutations, we performed ultra-high depth full mtDNA sequencing using overlapping primer pairs to amplify and sequence mtDNA from hearts, tibialis anterior muscles, livers and spinal cords of WT, G58R and S59L mice (Fig. 1A). The WT and G58R mice had an age range of 37–107 weeks, whereas the S59L mice were between 39–44 weeks old, as they all die before 14 months of age. As expected, almost all of the deletions clustered in the major arc (Fig. 1B, Supplementary Fig. 1A and Supplementary File 1) [45]. Upon quantification of deletion levels, we found that G58R mice had higher deletion total burdens compared to their WT littermates in the heart, tibialis and liver but not the spinal cord (Fig. 1C and Supplementary Fig. 1B). These tissues also showed a significant trend of increased mtDNA deletions with age in G58R mice. S59L mice also had higher deletion total burdens compared to WT in the heart and tibialis, but not liver or spinal cord. Remarkably, deletion levels were overall much lower in the spinal cord compared to the other three tissues for all three genotypes, indicating that spinal cord is resilient against developing mtDNA deletions both in WT and Chchd10 mutant mice.

**Figure 2:**
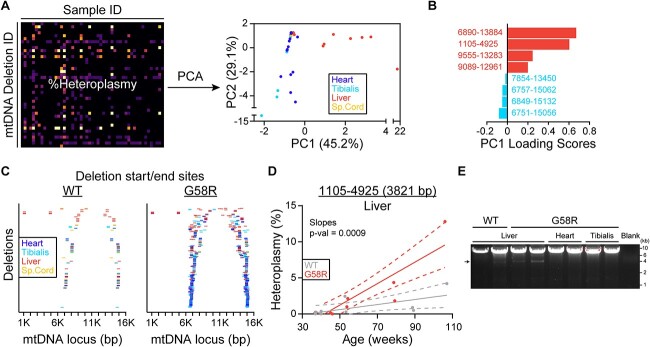
The G58R mutation accelerates the accumulation of a liver deletion. (A) Left: Demonstration of the matrix setup used for PCA. Right: PCA for all samples with at least 3 identified deletions. The numbers within the parentheses indicate the percentage of data variance that is explained by each PC. (B) The deletions with the highest (liver predominance) and lowest (heart/muscle predominance) PC1 loading scores. (C) Start and end breakpoints of all the deletions identified from WT and G58R mouse tissues. Each row is an individual deletion, with deletions arranged by size. (D) Heteroplasmy vs. age plot of the 3821 bp deletion in liver samples of WT and G58R mice. (E) Long-range PCR amplifying an ~8 kb region surrounding the minor arc. The bright band around 8 kb is full-length mtDNA, and the fainter band indicated by an arrow is the 3821 bp deletion. For (D) an equality of slopes test was performed.

We then wondered whether it was possible to find an overall deletion signature in an unbiased manner. To do this, deletions for each sample with at least 3 different deletions were arranged in matrix format and a principal component analysis (PCA) was performed (Fig. 2A). Indeed, we found that heart/tibialis and liver samples were separated by PC1, with heart and tibialis clustering together on the lower end of PC1, and liver samples spreading at the higher ranges of PC1. We additionally calculated the loading scores for PC1 to identify the specific deletions driving the samples towards the lower or higher ends of PC1 (Fig. 2B and Supplementary Fig. 2A). Looking at the distribution of these deletions across different tissue types confirmed their tissue specificity (Table 1). The two outlier samples from the PCA (one liver and one tibialis) both came from the oldest G58R mouse, as they had the highest levels of their respective tissue-specific deletions and were thus driven far along their respective PC axes (Fig. 2A). Of note, one of the positive drivers of PC1 (marking liver samples) was a 3821 bp minor arc deletion (Δ1105–4925) that was present almost exclusively in liver samples, except for one G58R heart sample where it was present at a very low heteroplasmy fraction (0.58%) (Fig. 2C). This mutation was previously identified as a naturally occurring age-related deletion in mouse livers [6]. We also found that the heteroplasmy fraction of this deletion increases with age in WT liver samples (Fig. 2D). Interestingly, the rate of accumulation of this deletion was faster in G58R livers. We validated the presence of this deletion in livers and its absence in other tissue types by long-range PCR of the minor arc (Fig. 2E). When examining another deletion that was a negative driver of PC1 (marking heart and tibialis samples), we found again that its rate of accumulation was prominently accentuated by the G58R mutation (Supplementary Fig. 2B). Thus, the G58R mutation accelerates the formation of two naturally occurring deletions.

**Table 1:** All deletions identified in at least 7 samples.

Deletion (bp)	Heart	Tibialis	Liver	Spinal Cord	WT	G58R	S59L	Total
7215–14 913	13	7	8	5	13	14	6	33
7287–14 938	11	9	8	4	9	21	2	32
8100–14 918	6	9	8	6	15	11	3	29
7393–13 936	8	5	2	2	5	12	0	17
6757–15 062	10	5	0	0	0	15	0	15
6849–15 132	9	5	1	0	0	15	0	15
6751–15 056	7	5	1	0	0	13	0	13
8948–14 260	2	0	10	0	4	8	0	12
6890–13 885	1	1	5	3	1	9	0	10
1105–4925	1	0	8	0	3	6	0	9
9555–13 283	1	1	6	0	2	6	0	8
7073–14 790	3	1	2	2	4	2	2	8
6628–12 882	5	2	0	0	0	7	0	7
6889–14 938	4	1	2	0	0	7	0	7
6890–13 884	4	0	3	0	0	7	0	7
7854–13 450	2	5	0	0	2	5	0	7
9089–12 961	1	1	5	0	2	5	0	7

The number of samples in which each deletion was identified is indicated. E.g., deletion 1105–4925 was identified in 9 total samples (1 heart and 8 livers; from 3 WT and 6 G58R samples).

**Figure 3:**
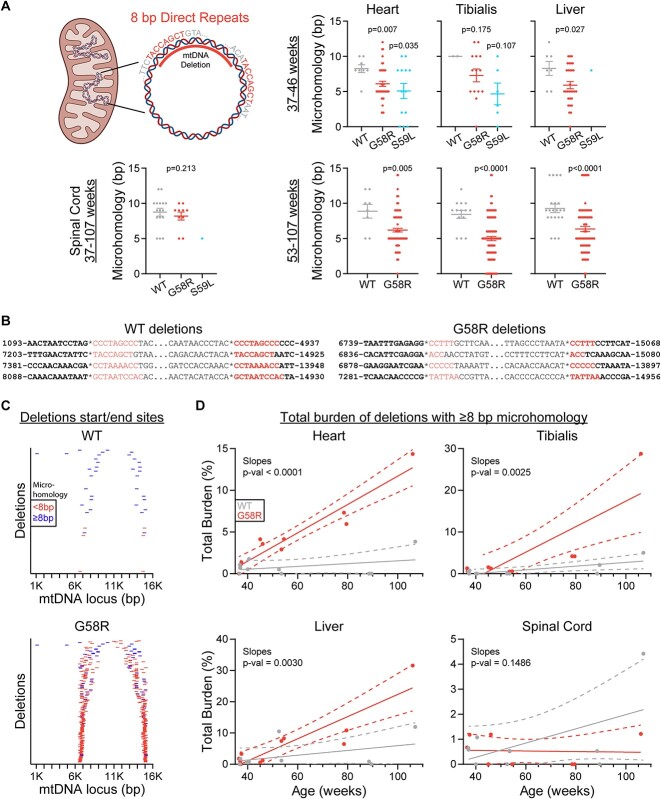
Chchd10 mutant mtDNA deletions are flanked by shorter direct repeats. (A) Top left: Illustration of an 8 bp direct repeat (microhomology) present in two locations within the mitochondrial genome. Note how one of the repeats is within the range of the deletion and is therefore lost in the deleted molecule. Rest: Length of direct repeats surrounding each deletion. (B) Representative WT and G58R mtDNA deletions. Direct repeats are coloured red, and deletion breakpoints are marked by asterisks. (C) Start and end breakpoints of all the unique deletions identified and coloured by the length of the direct repeats flanking them. Each deletion is shown only once even if it was identified in multiple samples. (D) Total deletion burden vs age plots for samples of the respective tissues, counting only deletions with microhomologies of 8 bp or longer. For (A), one-sided Mann-Whitney tests were performed. For (C), equality of slopes tests were performed.

We then asked whether Chchd10 mutations simply accelerate some naturally occurring deletions or also lead to the formation of new deletions through a different mechanism altogether. To test this, we assessed direct repeats in the immediate vicinity of the breakpoints for each deletion identified. Intriguingly, in all tissues except for the spinal cord, both G58R and S59L deletions tended to be flanked by shorter direct repeats compared to WT (Fig. 3A and B). When looking at all unique deletions identified, it became clear that many deletions specific to G58R mice have direct repeats fewer than 8 bp in length (Fig. 3C). Therefore, the G58R mutation dramatically increases the population of deletions flanked by shorter direct repeats. We next wondered whether this mutation also affected the total burden of deletions with direct repeats of 8 bp or more. We therefore looked at the total burden of all deletions flanked by direct repeats of at least 8 bp in all of the samples and found that the G58R mutation accelerated the rate of development of these mutations in all tissues examined except for the spinal cord (Fig. 3D). Thus, the G58R mutation both accelerates the formation of naturally occurring deletions with direct repeats of at least 8 bp, as well as leads to the formation of novel deletions with shorter microhomologies, indicating a different mechanism to those formed naturally with age.

We previously found decreased mtDNA CN in ~40-week old G58R mouse hearts [36], so we directly measured mtDNA CN in the genomic DNA of the sequenced samples. In heart and tibialis, both G58R and S59L mice had decreased mtDNA CN across all ages, whereas only G58R mice had decreased liver mtDNA CN (Supplementary Fig. 3). Similar to the absence of a deletion phenotype in spinal cord mtDNA, Chchd10 mutant spinal cords did not have decreased mtDNA CN compared to their WT littermates.

**Figure 4:**
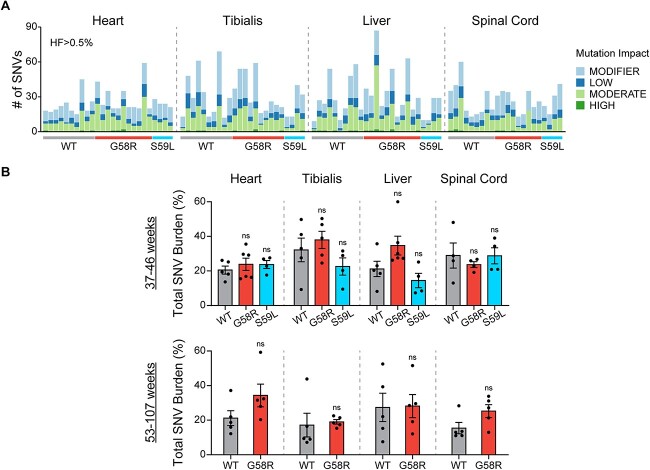
Chchd10 mutations do not increase mtDNA SNV mutational burdens. (A) Number of SNVs identified for each sample sequenced, annotated by impact of the mutation. (B) Summed SNV heteroplasmy fractions (expressed as percentages) for each sample, with samples stratified into two age groups. For (B), two-sided Mann-Whitney tests were performed.

We finally tested whether Chchd10 mutations affected mtDNA SNV accumulation. After calling variants and classifying them by impact, we did not find a difference between SNV burden or total heteroplasmy fractions between WT and Chchd10 mutant mice, indicating that while Chchd10 mutations cause mtDNA deletions, they do not increase SNV mutation load (Fig. 4A and B and Supplementary File 2).

## Discussion

The effect of Chchd10 mutations on the mtDNA deletion and mutational landscape had not been previously studied in depth. Here, using knockin mouse models of human disease-causing Chchd10 G58R and S59L mutations, we found that both mutations increased mtDNA deletion burden in heart and skeletal muscle in an age-dependent manner, but the spinal cord was resilient against accumulating deletions in both mutants. In addition to accelerating the accumulation of naturally occurring deletions which tend to be flanked by direct repeats of 8 or more bases, Chchd10 mutations also led to the development of novel deletions with shorter direct repeats. Finally, although Chchd10 mutations cause multiple mtDNA deletion syndromes, we found that in mice they do not lead to increased mtDNA SNV mutational burdens.

**Figure 5:**
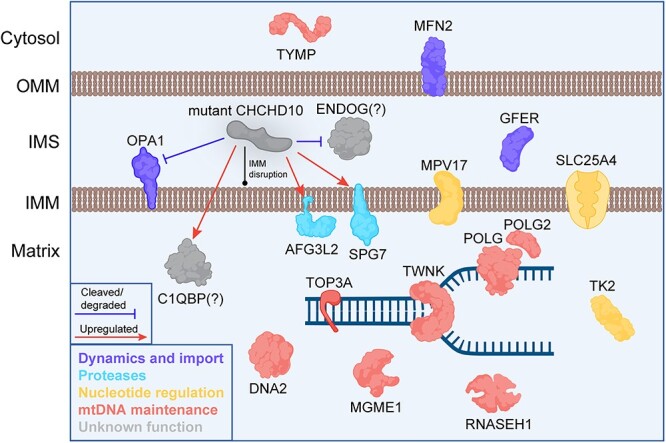
Proteins implicated in multiple mtDNA deletion syndromes, and known effects of CHCHD10 mutations on some of these proteins. Mutant CHCHD10 causes increased levels of C1QBP, AFG3L2 and SPG7 and cleavage or degradation of OPA1 and ENDOG [36]. In addition, CHCHD10 mutations disrupt IMM stability. These changes could contribute towards mtDNA deletion formation in CHCHD10 mutants.

It is not immediately clear how mutations in Chchd10, an IMS protein, can lead to instability of mtDNA in the matrix (Fig. 5). We previously showed strong activation of the inner mitochondrial membrane (IMM) protease OMA1 in G58R tissue, leading to the cleavage and depletion of the long forms of OPA1 necessary for fusion of the IMM [36]. Since loss of function mutations in OPA1 are known to cause multiple mtDNA deletions, it would be reasonable to assume that the increased cleavage of OPA1 caused by the G58R mutation would be contributing to the formation of deletions. However, when we previously crossed the G58R mice with OMA1 KO mice we found that Chchd10^G58R/+^; OMA1^−/−^ mice surprisingly had even greater levels of deletions, despite OPA1 not getting cleaved [36]. Thus, OPA1 cleavage was protective against and not causative of the deletions. In our previous mitochondrial proteomics analysis of G58R vs WT mouse hearts, we found that the most decreased protein in G58R hearts was endonuclease G (Endog), a DNA nuclease located in the IMS and previously implicated in removing paternal mtDNA and translocating to the cytosol during apoptosis [36, 46, 47]. Since our previous proteomics was of the mitochondrial fraction, the decrease in Endog could have been explained by translocation to the cytosol in apoptotic cells. To briefly address this possibility, we confirm here that Endog is decreased both in the cytosol and mitochondria of Chchd10 mutant mouse hearts, thus eliminating the possibility that it is simply exiting mitochondria during apoptosis (Supplementary Fig. 4). Additionally, a case report of a patient with biallelic ENDOG mutations described a patient who had a myopathy with multiple mtDNA deletions in muscle, similar to our proband [36, 48]. On the other hand, Endog’s IMS localisation and the absence of a mtDNA deletion phenotype in livers of Endog KO mice are plausible arguments against its involvement in the mechanism of mtDNA deletion formation in Chchd10 mutant mice (although that study analysed deletions with a southern blot, which is less sensitive than long-range PCR) [49].

Although CHCHD10 is located in the IMS, it is known to interact with the matrix protein C1QBP (also called p32), mutations in which were recently reported to be associated with multiple mtDNA deletions [50–54]. It is therefore possible that *CHCHD10* mutations cause deletions by affecting C1QBP function. Additionally, we previously demonstrated that mutations in the IMM proteases AFG3L2 and SPG7 cause mtDNA instability and multiple mtDNA deletions [55, 56]. These proteins were also found to be upregulated in our G58R mice [36]. It is possible that this is a compensatory upregulation, as *CHCHD10* mutations severely disrupt the IMM and its complexes [36, 57]. Furthermore, mitochondrial nucleoids tend to be anchored to the IMM and are influenced by its dynamics [58, 59]. Thus, CHCHD10 could also be causing mtDNA deletions by disrupting IMM structure and nucleoid stability.

Previous studies in PolG mutant and Twinkle mutant mice, as well as in humans with pathogenic *POLG* variants, have found that mtDNA deletions in these mutants tend to be flanked by shorter or no direct repeats compared to deletions that arise naturally with age [10, 60, 61]. A similar pattern was observed for deletions caused by the introduction of DSBs in mtDNA [11]. Our findings place CHCHD10 mtDNA deletion syndromes within the spectrum of deletion syndromes with decreased length of direct repeats, suggesting the possibility of a shared mechanism of deletion formation such as DSBs or a slipped strand mispairing mechanism. There seems to exist a loose threshold of around 8 bp of microhomology, at or above which most naturally occurring deletions form, and this threshold is lowered in Chchd10 and other mutants. Indeed, we found that naturally occurring deletions still happen in Chchd10 mutant mice and accumulate at an accelerated rate compared to WT mice. Nonetheless, it remains to be determined whether these longer microhomology deletions are generated through the same mechanism as those flanked by shorter direct repeats.

We found that the spinal cord was especially resistant to the accumulation of mtDNA deletions, even in comparison to other predominantly post-mitotic tissues like striated muscle. Spinal cord involvement is not part of the phenotypic spectrum of the G58R mutation [36], and even S59L mice have minimal spinal cord motor neuron loss later in life [42, 43]; therefore, the absence of deletions cannot be explained by the death of cells that have high levels of deletions. Even the deletions present in G58R spinal cord seemed to be naturally occurring deletions, as their flanking direct repeats tended to be 8 bp or longer. A previous single-cell study showed increased mtDNA deletions in spinal cord neurons of ALS patients [62]. Although Chchd10 is highly expressed in motor neurons of the spinal cord, it is expressed at relatively low levels in bulk spinal cord compared to heart, skeletal muscle and liver [54]. Thus, it is possible that single-cell analysis of spinal cord motor neurons might reveal increased mtDNA rearrangements in these cells.

It is notable that S59L mouse livers did not display increased mtDNA instability while G58R livers did. This is consistent with our previous finding that an OMA1 stress response is activated in G58R but not S59L mouse livers, and consistent with a recent report by another group which did not find liver abnormalities in S59L mice [36, 63]. This suggests that G58R mice may have liver pathology, but this needs to be assessed functionally as liver dysfunction was not reported in G58R patients [33, 36].

Finally, we found that Chchd10 mutant tissues which accumulate deletions also tend to have decreased mtDNA CN. Thus, the decreased overall CN and increased deletion rates in tandem exacerbate the lack of full-length mtDNA, possibly contributing to the disease phenotypes especially later in life. In addition, since the decrease in copy number was only ~2-fold, it is unlikely that the increased heteroplasmy fractions of these deletions with age are being driven by a somatic bottleneck leading to the rapid segregation of pre-existing deletions as proposed in other contexts [44].

Our study has a few limitations. As with many mitochondrial disorders, there is some degree of phenotypic heterogeneity in humans with CHCHD10 mutations. In the large p.S59L family, there was a highly variable clinical presentation, with the only common element being a mitochondrial myopathy with multiple mtDNA deletions [35]. This was recapitulated by the S59L mouse model. The affected family members additionally showed varying degrees of neuronal involvement. Although S59L mice can live up to 60 weeks, the S59L tissue used was from 40–44 week-old mice, and we may be missing more severe mtDNA alterations occurring at the end-stage. The premature death caused by cardiomyopathy in S59L mice prevented the detection of age-related phenotypes such as more pronounced neuronal involvement. This should be taken into consideration when interpreting our spinal cord results. As for the p.G58R mutation, there are currently two known p.G58R families, both of whose affected members present with generalised myopathy [33, 36]. However, while all affected members of the UK family also have cardiomyopathy and die before 35 years of age, affected members of the Puerto Rican family do not show signs of cardiomyopathy and seem to have normal lifespans. Importantly, G58R mice recapitulate key elements of the disease such as myopathy, cardiomyopathy, mitochondrial cristal dilations and OMA1 activation. Therefore, although our mice do not directly match the phenotypes of specific human pedigrees, as a group they capture a range of relevant phenotypes thus allowing us to explore the correlation between mtDNA mutations and phenotypes in CHCHD10 diseases.

Additionally, our sequencing set-up used two overlapping primer pairs to amplify the major and minor arcs separately. It is therefore possible that we are missing some deletions that include the primer binding sites, although most previously reported deletions are between the two primer pairs we used. PCR of mtDNA could also introduce amplification bias, favouring the amplification of shorter (deleted) templates. However, since the post-PCR heteroplasmy fraction nevertheless correlates with the starting heteroplasmy fraction [64], this method can still be used in a semi-quantitative manner. Therefore, the total deletion burdens we report are suitable for internal comparison of the samples and for inferring trends in deletion accumulation with age or genotype, rather than for obtaining absolute heteroplasmy fractions of deleted mtDNA for the samples. When interpreting the trends data, it should also be noted that there was only one animal of each genotype at the >100-week-old timepoint, which could have a disproportionate influence on the trend. Finally, human tissue was unfortunately not available for sequencing; it will be interesting to see if the same deletion signature is present in humans.

In summary, we sequenced mtDNA obtained from various tissues of WT and *Chchd10* mutant mice to characterise the mtDNA deletion signatures of the mutant lines. The deletion levels were higher in G58R and S59L mice than in WT mice in some tissues, and the deletion burden increased with age. We also found that the spinal cord was more resistant to the development of mtDNA deletions than the other tissues examined. Finally, in addition to accelerating the rate of naturally occurring deletions, *Chchd10* mutations also led to the accumulation of a unique set of deletions distinguished by shorter direct repeats flanking their breakpoints. This study therefore provides the first in-depth examination of the landscape of mtDNA alterations caused by *Chchd10* mutations in mouse, which may also be contributing to the clinical phenotype of *CHCHD10* pathogenic mutations in humans.

## Materials and methods

### Mouse models

Mice were maintained on a 12-h light/12-h dark cycle, with food and water provided ad libitum. Chchd10^G58R^ and Chchd10^S59L^ mice were previously generated as described earlier [36, 41]. All animal studies were approved by the Animal Care Use Committee of the NINDS, NIH intramural program.

### Tissue harvest and DNA extraction

Mice were anesthetised with isoflurane and transcardially perfused with PBS. Hearts, tibialis anterior muscles, livers and the lumbar enlargements of spinal cords were collected and immediately flash-frozen in liquid nitrogen. The lumbar region of the spinal cord was chosen because motor neuron loss was previously reported in that region in Chchd10^S59L^ mice [42]. Whole DNA was extracted from 15–25 mg of tissue using the DNeasy Blood and Tissue Kit (QIAGEN, cat# 69504) according to the manufacturer’s instructions. This yields gDNA and mtDNA which can be both sequenced and assessed for mtDNA CN. In total, 25 hearts, 24 tibialis anteriors, 25 livers and 22 spinal cords were obtained from 11 Chchd10^WT^, 12 Chchd10^G58R^ and 4 Chchd10^S59L^ mice. Specific ages and mouse IDs of collected tissues can be found in the Supplementary Files.

### Long-range PCR

mtDNA was amplified in two separate reactions using the following overlapping primer pairs: 5′-AGCAAAAGCCCACTTCGCCA-3′ and 5′-GGTTGGCCCCCAATTCAGGT-3′ for the major arc, and 5′-ACCTGAATTGGGGGCCAACC-3′ and 5′-TGGCGAAGTGGGCTTTTGCT-3′ for the minor arc [65]. The final PCR reaction contained 50 ng of starting DNA, 1X PrimeSTAR GXL Premix (TaKaRa, cat# R051A) and primers at a concentration of 200 nM each. The following settings were used: 94°C for 1 min, 30× (98°C for 10 s, 62°C for 30 s, 68°C for 10 min) and an infinite hold at 4°C. 5 μl of the PCR product was diluted thrice in dH_2_O and run on a 0.7% agarose gel precast with SYBR Safe (Invitrogen, cat # S33102), and finally imaged with a Gel Doc XR+ (Bio-Rad) to ensure the presence and proper length of DNA. The remaining 20 μl of the PCR product was used for sequencing.

### Sequencing of mtDNA

20 μl of PCR product was purified with 36 μl of AMPure XP beads (Beckman Coulter, cat# A63880). Concentration of the purified DNA was then measured with the Quant-iT dsDNA BR kit (Invitrogen, cat# Q33130). The PCR product was normalised to 10 ng/μl and 13 μl each of the major and minor arc PCR products were mixed together to yield a 26 μl sample. Library preparation was then performed on 96 samples using the NEBnext Ultra II FS Library Prep Kit for Illumina (NEB, cat# E7805L), NEBnext adaptors (NEB, cat# E7600S) and AMPure XP beads according to the kit’s instructions. In short, fragmentation was performed for 5 min at 37°C then 30 min at 65°C, followed by adaptor ligation. Size selection to attain a 500 bp insert size was achieved by two sequential AMPure XP purifications with beads added at 0.7X then 0.6X volume. PCR enrichment of ligated DNA was performed for 30 s at 98°C, then 5 cycles of 98°C for 10 s and 65°C for 75 s and a final elongation step of 65°C for 5 min. The final purification was performed with an AMPure bead concentration of 0.55X. Sample quality and concentration was assessed using HSD5000 ScreenTapes (Agilent, cat# 5067-5592) and reagents (Agilent, cat# 5067-5593). Samples were diluted to 4 nM, and 5 μl of each sample was added to generate the library. The library was processed according to the instructions for the Illumina MiSeq Reagent Kit v3 (600 cycle) (Illumina, cat# MS-102-3003) for a final denatured pooled library concentration of 20 pM. 5 μl of 20 pM denatured PhiX library (Illumina, cat# FC-110-3001) was added to 595 μl of the denatured pooled library for the final product which was sequenced on the Illumina MiSeq system.

### Bioinformatic analyses

Sequencing reads were aligned to the mouse mitochondrial genome (NCBI37/mm9 and NC_005089 with mouse nuclear mitochondrial DNA segments hard-masked) using BWA-MEM (version 0.7.15-r1142-dirty). Deletions were called through MitoSAlt (version 1.1) using the following conditions: score_threshold = 80; evalue_threshold = 0.00001; split_length = 15; paired_distance = 1000; deletion_threshold_min = 30; deletion_threshold_max = 30 000; breakthreshold = −2; cluster_threshold = 5; breakspan = 15; sizelimit = 10 000; hplimit = 0.01; flank = 15; split_distance_threshold = 5 [45, 66]. The called deletions were then filtered for those with a heteroplasmy fraction of at least 0.5%, and impossible deletions were filtered out (i.e. deletions encompassing the primer sites at bases 6551–6573 or 15 150–15 169 of the mouse mtDNA genome), although these were minimal and had very low heteroplasmy fractions. Burden was defined as the number of deletions called for each sample, and total burden was defined as the sum of the heteroplasmy fractions (as percentages) of all the deletions called for each sample. The length of direct repeats for each deletion was obtained by counting the number of bases of the microhomology sequence provided by MitoSAlt, and if no such sequence was identified then a microhomology length of 0 was assigned to that deletion.

SNVs were called using VarScan (version 2.3.9) and filtered at 600X depth and 0.5% heteroplasmy thresholds. Homoplasmic variants were additionally filtered out. Annotation and pathogenicity prediction of mtSNVs were obtained by applying SnpEff (version 5.1).

### mtDNA copy number assessment

Extracted DNA was diluted in dH_2_O to 1 ng/μl (for actin) and 0.0125 ng/μl (for ND1/CO3) and 10 μl of diluted DNA was used per reaction. The final reaction volume was 22 μl including 1X ddPCR Supermix for Probes (No dUTP) (from 2X stock, Bio-Rad, cat# 186-3025), 400 nM forward and reverse primers and 225 nM probe(s). The primers used were actin (F: 5′-CTGCTCTTTCCCAGACGAGG-3′, R: 5′-AAGGCCACTTATCACCAGCC-3′, FAM probe: 5′-ATTGCCTTTCTGACTAGGTG-3′), ND1 (F: 5′-GAGCCTCAAACTCCAAATACTCACT-3′, R: 5′-GAACTGATAAAAGGATAATAGCTATGGTTACTTCA-3′, FAM probe: 5′-CCGTAGCCCAAACAAT-3′), CO3 (F: 5′-CCTCGTACCAACACATGATCTAGG-3′, R: 5′-AGTGGGACTTCTAGAGGGTTAAGTG-3′, HEX probe: 5′-ACCTCCAACAGGAATTTCA-3′). All probes had the quencher BHQ-2. Droplets were generated using the AutoDG Droplet Generator (Bio-Rad, cat# 1864101) and the PCR was performed as follows: 95°C for 10 min, 40× (94°C for 30 s ramp at 2°C/s, 58°C for 1 min ramp at 2°C/s) and 98°C for 10 min. Droplets were then analysed with the QX200 Droplet Reader (Bio-Rad, cat# 1864003). Absolute copy number was calculated as follows: mtDNA CN = $2\times \frac{\left[ ND1\ or\ CO3\right]}{\left[ Actin\right]}$ where ND1 or CO3 was chosen based on whichever was higher for the respective sample, to account as much as possible for major and minor arc deletions.

### Subcellular fractionation and western blotting

Isolation of the cytosolic and mitochondrial fractions and western blotting were performed as previously described [36]. The antibody used was Endog (Santa Cruz, cat# sc-365359).

## Supplementary Material

Supplementary_Figures_ddad161

SuppFigs_Revision_ddad161

Supplementary_File_1_mtDNA_deletions_ddad161

Supplementary_File_2_mtDNA_SNVs_ddad161

Supplementary_figure_legends_ddad161

## Data Availability

Raw sequencing data was deposited to NCBI's Sequence Read Archive (BioProject ID PRJNA1020161), and the processed data is available in Supplementary Files 1 and 2.
