# Supplementary material for: CHCHD10 mutations induce tissue-specific mitochondrial DNA deletions with a distinct signature
Source: Hum Mol Genet. 2023 Oct 10;33(1):91–101. doi: 10.1093/hmg/ddad161 (PMC10729859; doi:10.1093/hmg/ddad161)
Supplement: Supplementary_Figures_ddad161 [file supplementary_figures_ddad161.zip › Supplementary_Figures_ddad161.pdf]

Supplementary Figures

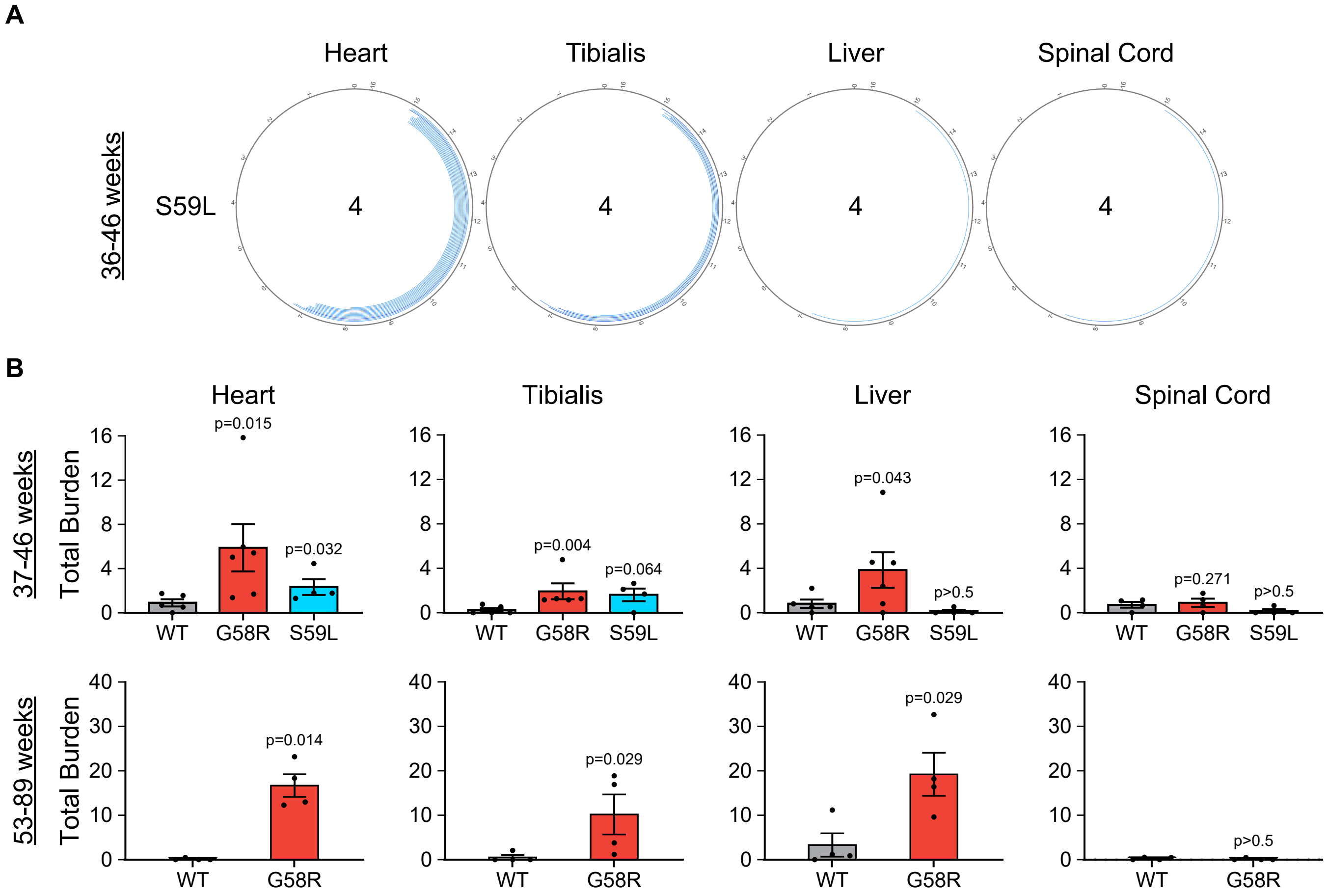

Figure S1. Quantification of identified deletions. (A) Circle plots representing mtDNA and showing the deletions called through MitoSAlt for WT, G58R and S59L mice. Darker shades correspond to higher heteroplasmy (range: 0.5% - 2.1%). The darker the shade of blue the higher the heteroplasmy of that deletion. Deletions shown are compiled from the number of samples indicated in the centre of the circle. (B) Aggregated data from Fig. 1C. For (B), one-sided Mann-Whitney tests were performed.

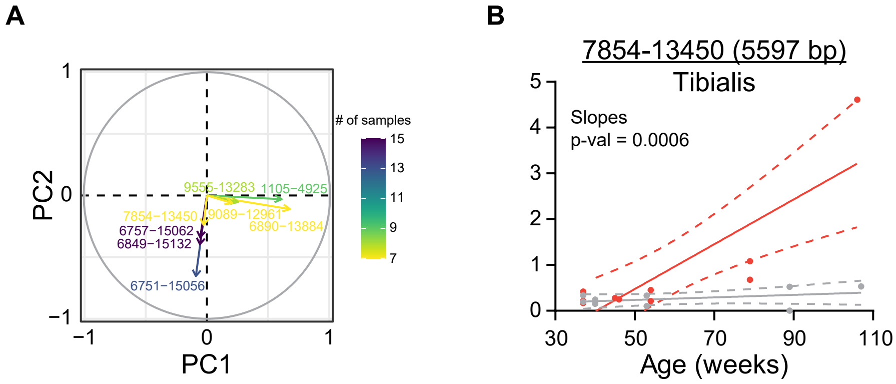

Figure S2. Deletions in different tissues. (A) PC1 and PC2 loading scores of the 4 deletions with the highest and 4 deletions with the lowest PC1 scores. (B) Heteroplasmy vs. age plot of the 5597 bp deletion in tibialis samples of WT and G58R mice. For (B), an equality of slopes test was performed.

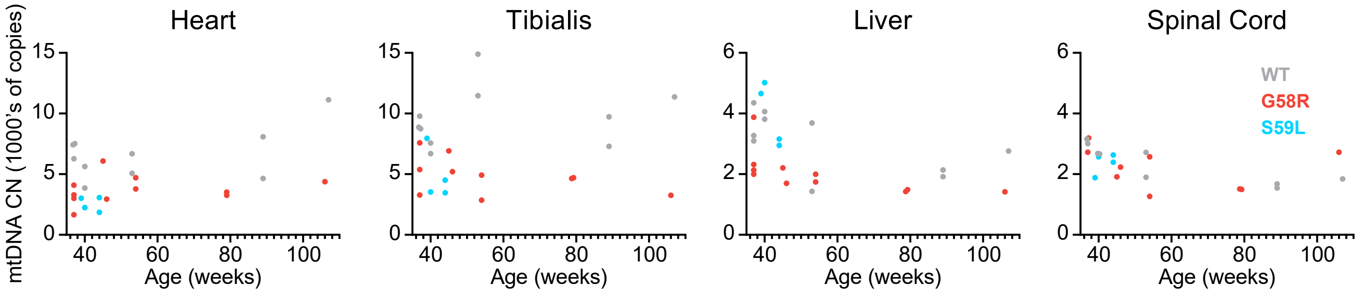

Figure S3. mtDNA copy number assessment.

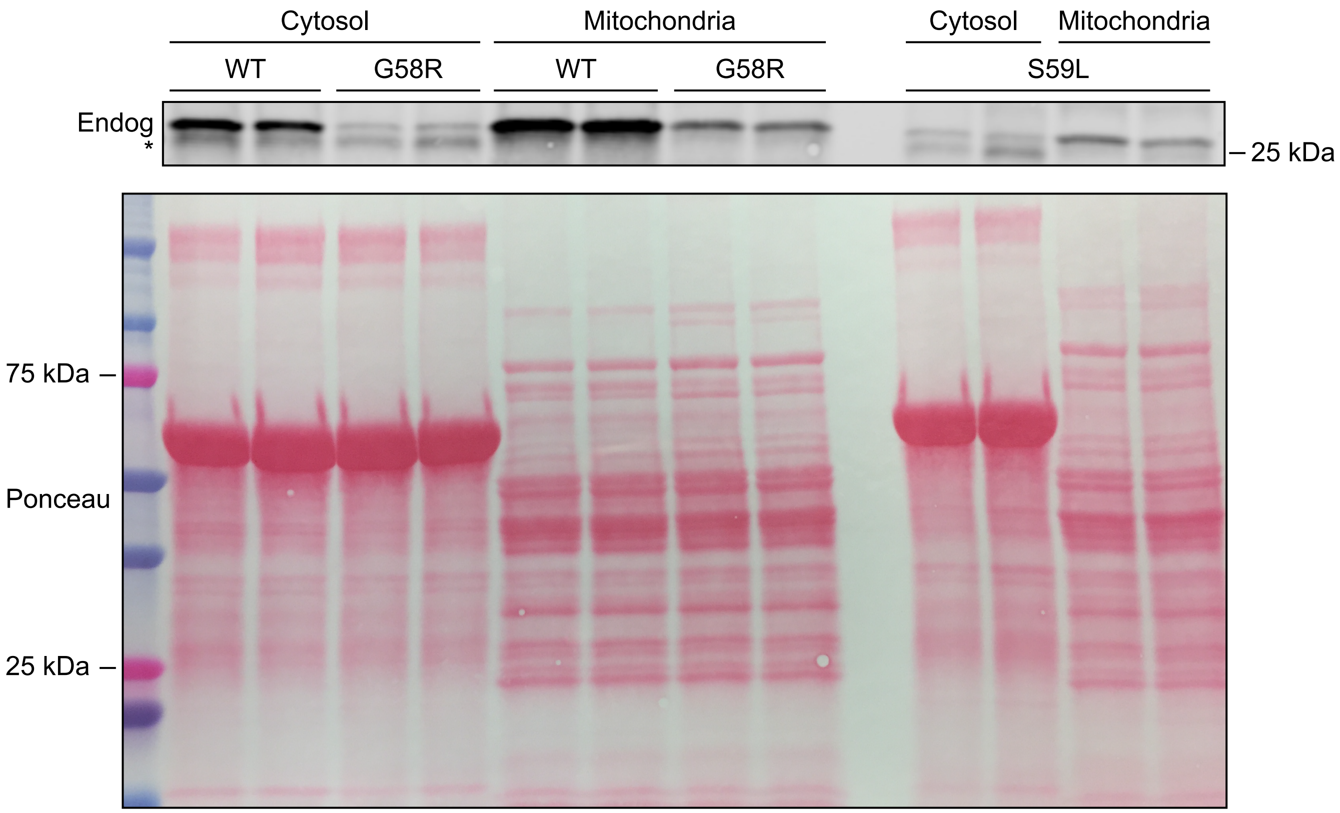

Figure S4. Western blot of Endog from mouse heart cytosolic and mitochondrial fractions. Two mice were used per genotype. The bottom blot displays the Ponceau stain from the respective lysates.
