# Supplementary material for: CHCHD10 mutations induce tissue-specific mitochondrial DNA deletions with a distinct signature
Source: Hum Mol Genet. 2023 Oct 10;33(1):91–101. doi: 10.1093/hmg/ddad161 (PMC10729859; doi:10.1093/hmg/ddad161)
Supplement: Supplementary_figure_legends_ddad161 [file supplementary_figure_legends_ddad161.docx]

**Supplementary figure legends**

**Figure S1. Quantification of identified deletions.** (**A**) Circle plots representing mtDNA and showing the deletions called through MitoSAlt for WT, G58R and S59L mice. Darker shades correspond to higher heteroplasmy (range: 0.5% - 2.1%). Deletions shown are compiled from the number of samples indicated in the centre of the circle. (**B**) Aggregated data from Fig. 1C. For (**B**), one-sided Mann-Whitney tests were performed.

**Figure S2. Deletions in different tissues.** (**A**) PC1 and PC2 loading scores of the 4 deletions with the highest and 4 deletions with the lowest PC1 scores. (**B**) Heteroplasmy vs. age plot of the 5597 bp deletion in tibialis samples of WT and G58R mice. For (**B**) an equality of slopes test was performed.

**Figure S3. mtDNA copy number assessment.**

**Figure S4. Western blot of Endog from mouse heart cytosolic and mitochondrial fractions.** Two mice were used per genotype.
